# CD137 Regulates NFATc1 Expression in Mouse VSMCs through TRAF6/NF-*κ*B p65 Signaling Pathway

**DOI:** 10.1155/2015/639780

**Published:** 2015-10-27

**Authors:** Jinchuan Yan, Yunjie Yin, Wei Zhong, Cuiping Wang, Zhongqun Wang

**Affiliations:** Department of Cardiology, Affiliated Hospital of Jiangsu University, Zhenjiang, Jiangsu 212001, China

## Abstract

Our previous study proved that CD137-CD137L interaction can regulate the expression of NFATc1. Here, we investigated whether CD137 signaling regulates the expression of NFATc1 in mice VSMCs through TRAF6/NF-*κ*B p65 pathway. Data shows that the CD137 expression can be stimulated by TNF-*α* in a time-dependent manner in mouse VSMCs. Knockdown of TRAF6 by siTRAF6 significantly attenuated agonist-CD137mAb induced increase of NF-*κ*B p65 and NFATc1 in VSMCs. Pretreatment with a NF-*κ*B inhibitor PDTC for 30 min inhibited the expression of p-p65 in both cytoplasm and nucleus in VSMCs. Thus, the protein level of NFATc1 can be suppressed through inhibition of p-p65. Finally, we also show that the levels of IL-2 and IL-6 can be increased by agonist-CD137 stimulation and decreased when NFATc1 was suppressed. Our data suggest that activated CD137 signaling regulates the expression of NFATc1 and its downstream factors through TRAF6/NF-*κ*B p65 pathways in VSMCs. These findings provide a novel target for treatment of atherosclerosis.

## 1. Introduction

Vascular inflammation plays an important role during the development and progression of atherosclerosis [1], which is the underlying etiology of cardiovascular diseases all over the world. However, the molecular mechanisms of atherosclerosis remain unclear. Both innate and adaptive immunities are involved in this disease [2]. CD137, a costimulating molecule, is found in the atherosclerotic plaques. Accumulating evidence suggests that CD137 signaling plays an important role in atherosclerosis [3, 4]. In our previous studies, we found that the level of soluble CD137 was significantly elevated in patients with acute myocardial infarction [5]. We also demonstrated that CD137-CD137L interactions can promote the progression of atherosclerosis [6]. CD137 signaling may lead to leukocyte recruitment and increased inflammation, which is closely related to atherosclerosis [3]. The nuclear factor of activated T cells (NFAT), an important transcription factor, plays a role in inflammation, calcification, and smooth muscle differentiation via regulation of smooth muscle-specific markers [7, 8]. NFATc1 is a hyperphosphorylated cytosolic protein that activates calcineurin which in turn dephosphorylates multiple serine residues and allows NFAT to be translocated into the nucleus [9]. Recent studies show that NFATc1 may promote inflammation in the progression and regression of atherosclerosis [10]. Our previous study proved that CD137-CD137L interaction can regulate the expression of NFATc1 in spleen lymphocyte in ApoE^−/−^ mice [11]. However, the specific factors that transmit the signal from CD137-CD137L to NFATc1 in mouse VSMCs remain unclear.

A recent study reported that TRAF6/NF-*κ*B/NFATc1 signaling may play an important role in osteoclast differentiation and bone resorption [12]. NF-*κ*B is one of most important transcription factors in inflammation. The NF-*κ*B family consists of five members (p50, p52, p65, c-Rel, and RelB). The dimer of p65-p50 is the inactivated form in the cytoplasm, and the inhibitor of NF-*κ*B (I*κ*B) is combined with the dimer to inhibit its activity. Upon activation by inflammatory cytokines, I*κ*B become degraded, resulting in the phosphorylation of NF-*κ*B p65 (p-p65). Subsequently, the p65-p50 dimer enters the nucleus and combines with DNA and then regulates the downstream gene expression [13]. This pathway can regulate many cell functions such as cell proliferation, inflammation, and apoptosis. TRAF6 is an important E3 ubiquitin ligase. TRAF6-mediated K63 ubiquitination is critical in mediating the activation of IKK and NF-*κ*B. Previous studies [14, 15] have shown that inhibition of the ubiquitination of TRAF6 can attenuate NF-*κ*B activation in cardiomyocytes and cardiac fibroblasts. Based on these studies, we hypothesized that TRAF6/NF-*κ*B p65 pathway may be involved in CD137 signaling induced expression of NFATc1 and its downstream factors expression in mice VSMCs.

## 2. Materials and Methods

### 2.1. Ethics Statement and Primary Cell Culture

The study protocol was reviewed and approved by the Animal Care and Use Committee of Jiangsu University. Eight-week-old male C57BL/6J mice were purchased from the animal center of Jiangsu University. Two C57BL/6J mice were executed by cervical dislocation, placed in 75% alcohol for 5 min, and fixed to the plate. The chest of the mice was then removed. The thoracic aorta was exposed via operation and observed under a surgical microscope. The artery was removed and washed with phosphate-buffered saline (PBS) two to three times. The artery was placed in type II collagenase at 37°C for 8 min, exposing the membrane fiber to remove the tunica adventitia. The vessels were cut into pieces and placed in cell culture flasks. Then, 5 mL of DMED/F12 (Hyclone) medium containing 20% fetal bovine serum (FBS) (Gibco) was added. Tissue blocks should not be made in DMEM. The sample was placed in the cell culture incubator at 37°C and 5% CO_2_. After 40 min, the tissue blocks can be made into DMEM carefully. The tissue blocks were removed after cells developed around them. The cells were stained with *α*-SMA (Sigma) to determine their purity.

### 2.2. Cell Treatment

At first, the expression of CD137 in VSMC was induced by 10 ng/mL TNF-*α* (Peprotech) for 24 h and analyzed by RT-qPCR and FCM; then agonist-CD137mAb (R&D) which could bind to the CD137 molecule was then used to stimulate these VSMCs to activate the CD137 signaling. TRAF6 siRNA (Ribobio) was transfected to knock down the expression of TRAF6 and the cells were incubated with agonist-CD137mAb for different time to collect NF-*κ*B protein (p65 and p-p65), as well as mRNA and proteins of TRAF6 and NFATc1. The siRNA and lipo2000 were diluted with Opti-MEM I prior to mixing. The siRNA and lipo2000 mixture was incubated for 20 min at room temperature and then added to the cell culture which contains 50 nM TRAF6. Incubation was continued for 24 h; then we tested the transfection efficiency. The levels of TRAF6 and NFATc1 proteins were measured using Western blot. The levels of TRAF6 and NFATc1 mRNA were detected by qRT-PCR. Cells were pretreated with PDTC (sigma) for 30 min, and then agonist-CD137mAb was added and incubated at 37°C, 5% CO_2_ for 90 min to collect NF-*κ*B protein and for 24 h to collect mRNA and protein of NFATc1. The level of NFATc1 protein was measured using Western blot. The level of NFATc1 mRNA was detected using qRT-PCR. PLKO.1-puro and sh-NFATc1 vectors were bought from Sigma. PLKO.1-shNFATc1 and PLKO.1-shCon were packaged into Lentivirus according to the protocol and infected VSMCs were infected with the Lentivirus to establish the stable cell line. The suppressed NFATc1 cells were stimulated with agonist-CD137mAb, and the supernatant was collected to detect the levels of IL-2 and IL-6 by ELISA.

### 2.3. Quantitative Real-Time PCR

RNA isolation and qRT-PCR were performed as previously described. Total RNA was isolated from VSMCs with Trizol-Reagent (Invitrogen) and then reverse transcribed using random hexamers and reverse transcriptase (Takara). cDNA was amplified using RT-PCR. Multiple mRNAs (Ct values) were quantified simultaneously using the software. The primers were synthesized from generay (Shanghai, China). The primer pairs were as follows: GAPDH: forward: GGC ATTGCTCTCAATGACAA, reverse: TGTGAGGGAGATGCTCAGTG, CD137: forward: CCTCCAAGTACCTTCTCC AGCA, reverse: CCTCCAAGTACCTTCTCCAGCA, TRAF6: forward: ACAAATACCTGAGGCAGTTCCCA, reverse: AAAGTACACGGACAAAATAGCCCA T, NFATc1: forward: TGGGAGATGGAA GCA AAG ACTGA, reverse: CATTGGCAGGAAGGTACGTGA A.

### 2.4. Western Blot Analysis

The cells were washed with PBS before being lysed in lysis buffer that can extract both cytoplasm proteins and nucleoproteins. The sample was stored at −80°C. Samples containing an equal amount of proteins were mixed with 5x SDS loading buffer and electrophoresed on a 10% SDS-PAGE gel. The proteins were then transferred onto PVDF membrane (Millipore). The membranes were blocked and probed with antibodies anti-NFAT2 (CST), TRAF6 (Immunoway), p-NF-*κ*Bp65 (Immunoway), PCNA (Immunoway), p65 (Immunoway), and *β*-actin (Proteintech group). The membranes were incubated with specific primary antibodies overnight at 4°C at a 1 : 1000 dilution. Subsequently, the membranes were washed with TBS/T for 15 min, three times, and incubated for 1 h with HRP-conjugated secondary antibodies (Abbkine). The protein was visualized with chemiluminescence, and a densitometric scanner was used to determine the density of the band. All experiments were repeated at least three times independently.

### 2.5. Enzyme-Linked Immunosorbent Assay

Cell culture supernatant levels IL-2 and IL-6 were measured using ELISA (Rapidbio). The cell culture supernatant was collected, and centrifugation was conducted for 20 min at the speed of 2000–3000 rpm. After centrifugation, the supernatant was removed. The sample was diluted and added to standard. The standard curve was drawn on the graph paper. For the assay procedure, the sample was added, incubated, liquid configured, and washed. Enzyme was then added, incubated, washed, and dyed, and the reaction and assay were terminated.

### 2.6. Flow Cytometry

FCM was used to detect the level of membrane CD137 in VSMC. The CD137 antibodies and isotype-matched control antibodies were diluted according to the instructions, with the final concentration of 1.25 *μ*g/mL. Cells were collected from different groups of VSMC. After washing by PBS, diluted CD137 antibody was added and incubated for 30 min in dark room at 4°C. After the incubation, the cells were washed again and then analyzed by Flow Cytometry (BD ACCURI C6).

### 2.7. Statistical Analysis

The results were expressed as means ± SD. All data were analyzed using SPSS 16.0.ANOVA, and *t*-test was used to compare differences among groups. *p* values less than 0.05 were considered statistically significant.

## 3. Results

### 3.1. TNF-*α* Stimulates CD137 Expression in VSMCs

**Figure 1:**
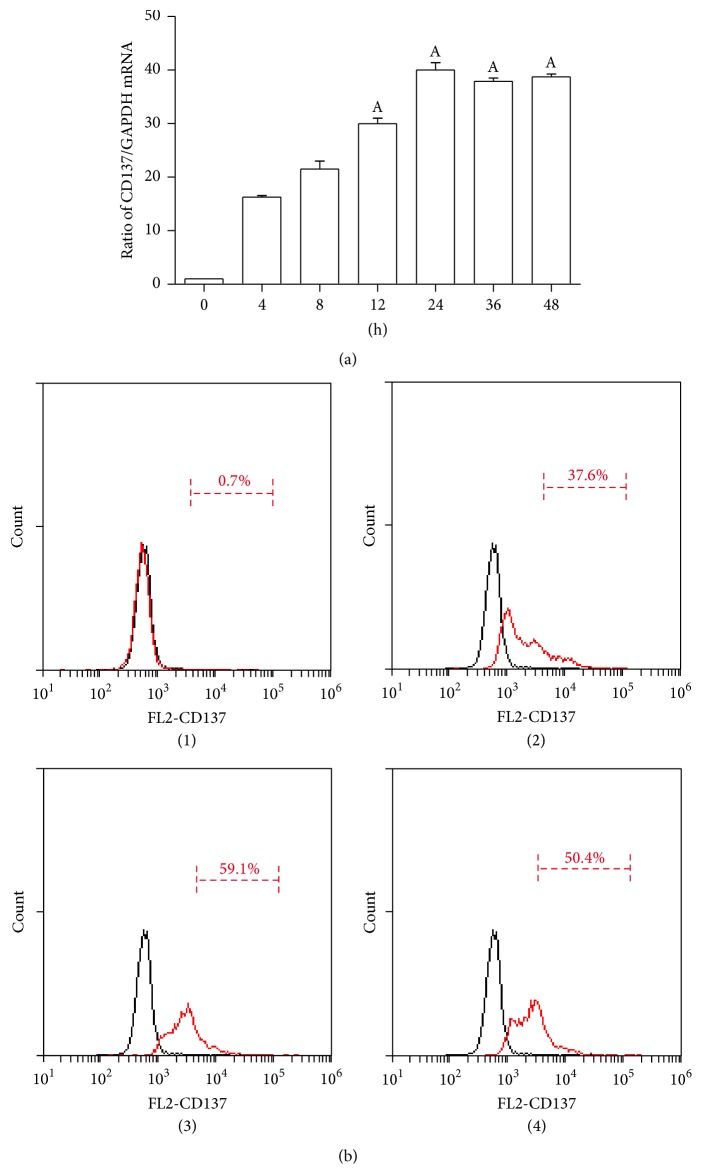
TNF-*α* stimulates CD137 expression in VSMCs. The VSMCs were treated with TNF-*α* (10 ng/mL) for different times. (a) The cells were harvested at 0, 4, 8, 12, and 24 h after treatment with TNF-*α* for examination of CD137 mRNA levels by qRT-PCR. (b) The expression of membrane CD137 was examined by FCM ((1) control, (2) stimulated 12 h by TNF-*α*, (3) stimulated 24 h by TNF-*α*, and (4) stimulated 36 h by TNF-*α*). ^A^ *p* < 0.05 versus control; *n* = 4.

Since the normal VSMCs do not express CD137, we first stimulated CD137 expression in VSMCs by TNF-*α* (10 ng/mL). Our data shows that CD137 mRNA expression can be stimulated by TNF-*α* in a time-dependent manner, which reached the peak at 24 h and was maintained at 48 h (Figure 1(a)). The FCM also shows that the expression of membrane CD137 in VSMCs was mostly induced by TNF-*α* at 24 h (Figure 1(b)).

### 3.2. TRAF6 Silencing Significantly Attenuated the Agonist-CD137mAb Induced Increase of NF-*κ*B p65 and NFATc1 in VSMCs

**Figure 2:**
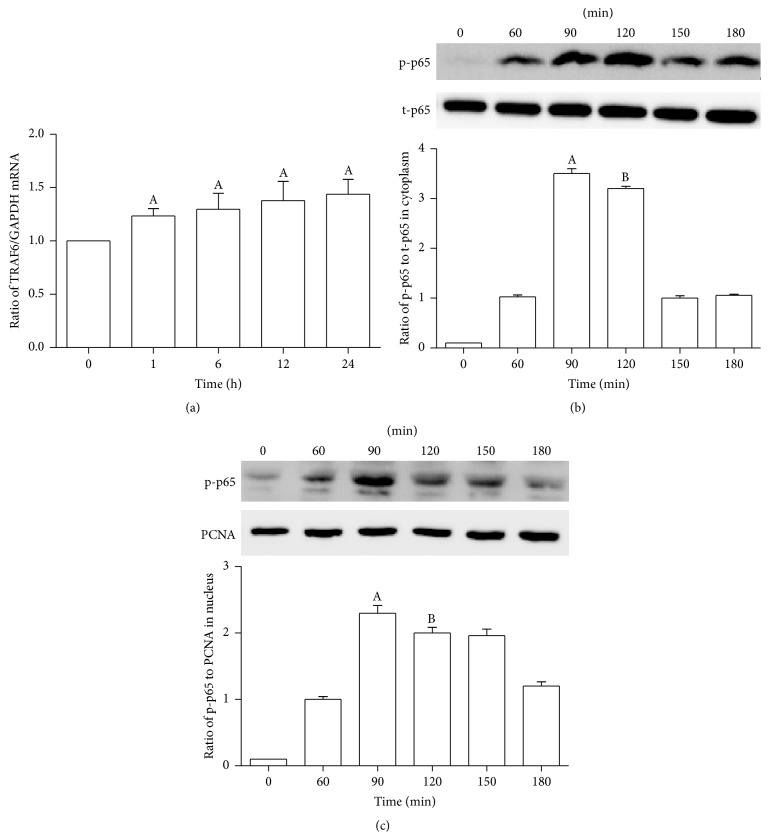
Agonist-CD137mAb could significantly increase the expression of TRAF6 and p-65 in VSMCs. The VSMCs were treated with agonist-CD137mAb (10 mg/L) for different times. (a) The cells were harvested at 0, 1, 6, 12, and 24 h after treatment with agonist-CD137mAb for examination of TRAF6 mRNA levels by qRT-PCR. (b, c) The cells were harvested at 0, 60, 90, 120, 150, and 180 min after treatment with agonist-CD137mAb. The nuclear and cytoplasmic proteins were isolated from the harvested cells. The protein levels of cytoplasm (b) and nucleus (c) were determined by Western blot. ^A, B^ *p* < 0.05 versus control; *n* = 4.

First, we activated the CD137 signaling by agonist-CD137mAb (10 *μ*g/mL). We found that the expression of TRAF6 and p-p65, two downstream factors of CD137 signaling, was significantly increased under the agonist-CD137mAb treatment.  Figure 2(a) shows that TRAF6 mRNA level was gradually increased in different time course (0, 1, 6, 12, and 24 h). And the protein levels of p-p65 were also increased at 60 min and reached the peak at 90 min both in cytoplasm (Figure 2(b)) and in nucleus (Figure 2(c)) by agonist-CD137mAb administration.

**Figure 3:**
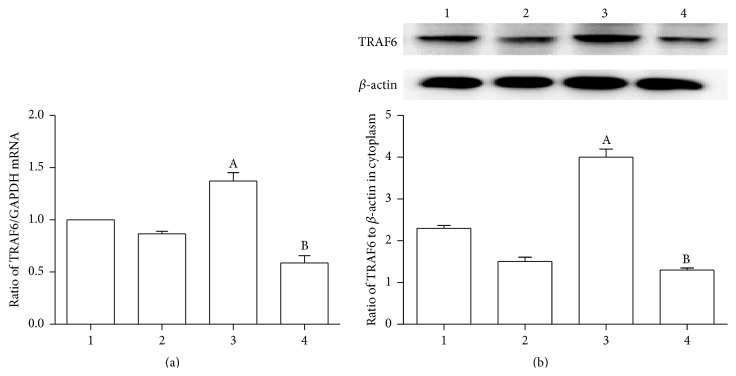
siTRAF6 could significantly attenuate the agonist-CD137mAb induced increasing of TRAF6 in VSMCs. The VSMCs were treated with agonist-CD137mAb (10 mg/L) for 12 h after transfection of siTRAF6. The cells were harvested for examination of TRAF6 mRNA (a) levels by qRT-PCR and protein (b) levels by Western blot. (1: control, 2: TRAF6siRNA, 3: agonist-CD137mAb, and 4: agonist-CD137mAb + TRAF6siRNA). ^A, B^ *p* < 0.05 versus control, ^B^ *p* < 0.05 versus CD137; *n* = 4.

To find out whether TRAF6 was involved in CD137-CD137L/NF-*κ*B/NFATc1 pathways, we silenced the expression of TRAF6 by treating VSMCs with siTRAF6. As shown in Figure 3, agonist-CD137mAb administration significantly increased both mRNA (Figure 3(a)) and protein (Figure 3(b)) levels of TRAF6, when compared with the control cells. However, transfection of siTRAF6 attenuated agonist-CD137mAb induced TRAF6 expression in VSMCs.

**Figure 4:**
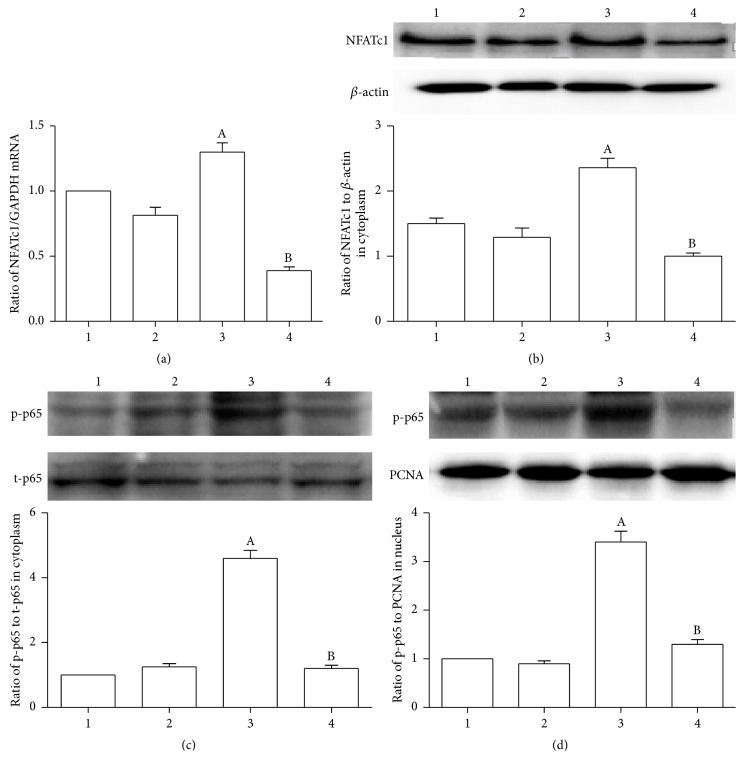
Silencing the expression of TRAF6 significantly attenuated the agonist-CD137mAb induced increasing of NF-*κ*B p65 and NFATc1 in VSMCs. The VSMCs were treated with agonist-CD137mAb (10 mg/L) for 12 h after transfection of siTRAF6. Total RNA and cytoplasm and nucleus protein were isolated from the harvested cells. The cells were harvested for examination of mRNA levels of NFATc1 (a) by qRT-PCR and protein levels of NFATc1 (b) and p-p65 cytoplasm (c) and nucleus (d) by Western blot. (1: control, 2: TRAF6siRNA, 3: agonist-CD137mAb, and 4: agonist-CD137mAb + TRAF6siRNA). ^A^ *p* < 0.05 versus control, ^B^ *p* < 0.05 versus CD137; *n* = 4.

Next, we examined the expression of p-p65 and NFATc1. We found that the agonist-CD137mAb increased p-p65 expression both in cytoplasm (Figure 4(a)) and in nucleus (Figure 4(b)) and was decreased by infection of siTRAF6 in VSMCs. Also the mRNA (Figure 4(c)) and protein (Figure 4(d)) levels of NFATc1 were significantly increased under agonist-CD137mAb treatment but decreased when transfected with siTRAF6.

### 3.3. Inhibition of NF-*κ*B Significantly Attenuated the Agonist-CD137mAb Induced Increase of NFATc1 in VSMCs

**Figure 5:**
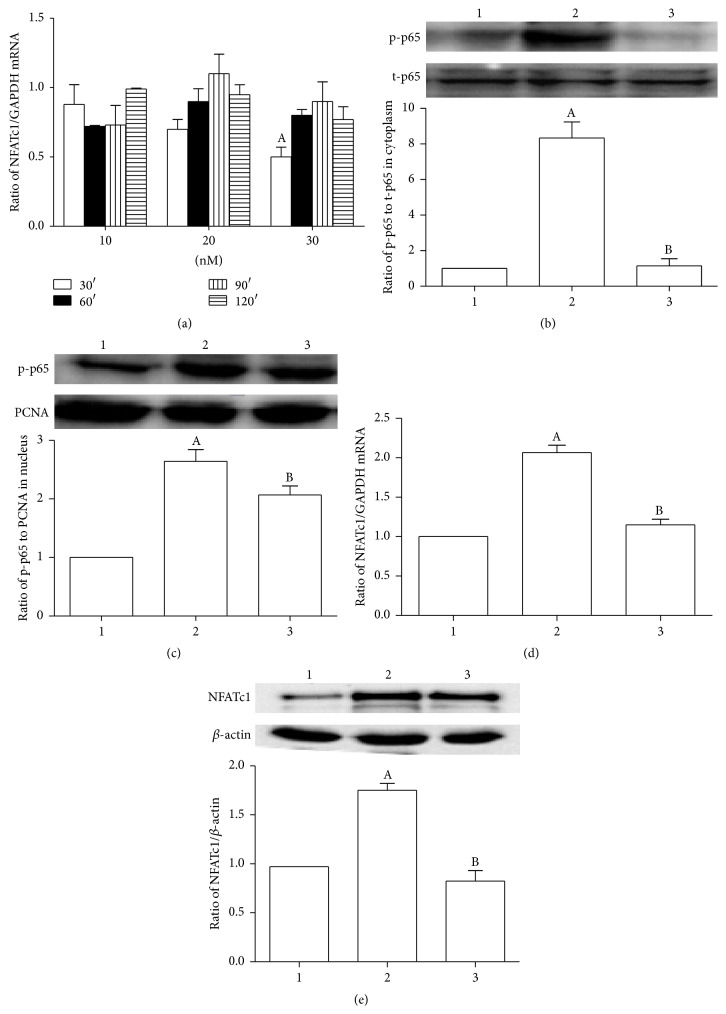
Inhibition of NF-*κ*B significantly attenuated the agonist-CD137mAb induced increasing of NFATc1 in VSMCs. The VSMCs were treated with PDTC (the specific inhibitor of the NF-*κ*B). (a) The cells were treated with 10 nM, 20 nM, and 30 nM PDTC for 30, 60, 90, and 120 min. Total RNA were isolated from the harvested cells. The mRNA levels of NFATc1 were determined by qRT-PCR. (b, c, d, e) The cells were pretreated with 30 nM PDTC for 30 min before agonist-CD137mAb administration. Total RNA and cytoplasm and nucleus protein were isolated from the harvested cells. The cells were harvested for examination of p-p65 cytoplasm (b) and nucleus (c) and protein levels of NFATc1 (e) by Western blot and mRNA levels of NFATc1 (d) by qRT-PCR. ^A^ *p* < 0.05; *n* = 4.

In order to find a more effective dose and time course, we treated the VSMCs with PDTC, a NF-*κ*B inhibitor, for different time and with various dose.  Figure 5(a) shows that pretreatment with 30 *μ*mol/L PDTC for 30 minutes was the most effective in inhibiting the expression of NFATc1. So we choose this dose and time course for the subsequent experiments. Our data shows that PDTC administration significantly decreased the expression of p-p65 both in cytoplasm (Figure 5(b)) and in nucleus (Figure 5(c)) of VSMCs. We then examined the effect of PDTC on the expression of NFATc1 in VSMCs. As shown in Figures 5(d) and 5(e), agonist-CD137mAb administration significantly increased both mRNA and protein levels of NFATc1, but PDTC attenuated the agonist-CD137mAb induced increasing of NFATc1.

### 3.4. TRAF6/NF-*κ*B/NFATc1 Suppressing Leads to Significant Inhibition of Agonist-CD137mAb Induced Secretion of IL-2 and IL-6 in VSMCs

**Figure 6:**
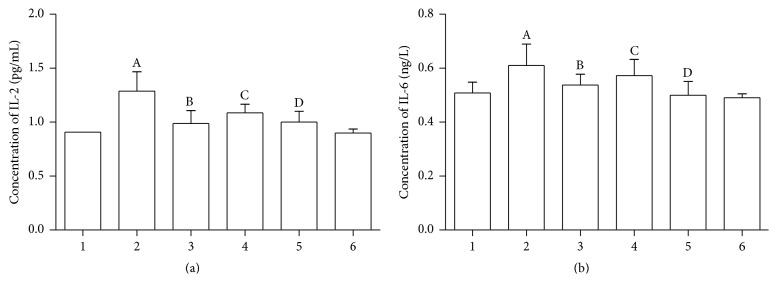
Suppressing TRAF6/NF-*κ*B/NFATc1 significantly attenuated the agonist-CD137mAb induced increasing of IL-2 and IL-6 in VSMCs. The VSMCs were treated with agonist-CD137mAb (10 mg/L) for 12 h after transfection of siTRAF6, pretreatment with PDTC, or using a stable cell line which suppressed NFATc1. The secretion of IL-2 (a) and IL-6 (b) was measured by ELISA (PLKO.1-shNFATc1 means the gene NFATc1 is silenced by Lentivirus infection, and PLKO.1-Con means negative control) (1: control; 2: CD137; 3: CD137mAb + siTRAF6; 4: CD137 + PTDC; 5: CD137 + PLKO.1-ShNFATC1; 6: PLKO.1-control). ^A, B, C, D^ *p* < 0.05 versus control; *n* = 4.

IL-2 and IL-6 were two well-recognized downstream factors of NFATc1. We therefore examined the role of TRAF6/NF-*κ*B/NFATc1 pathways on the expression of IL-2 and IL-6. Our data shows that agonist-CD137mAb administration significantly increased the expression of IL-2, while transfection of siTRAF6, pretreatment with PDTC or knockdown of NFATc1, all significantly attenuated the agonist-CD137mAb induced increase of IL-2 in VSMCs (Figure 6(a)). Also, Figure 6(b) shows that the expression of IL-6 was significantly increased under agonist-CD137mAb treatment but decreased when TRAF6 or NFATc1 is inhibited. Our data show that TRAF6/NF-*κ*B p65 pathway may be at least partially involved in CD137 signaling induced expression of NFATc1 and its downstream factors expression in mouse VSMCs.

## 4. Discussion

The results of our present study revealed several novel findings. CD137 expression was upregulated in TNF-*α* induced inflammatory mouse VSMCs. Silencing TRAF6 expression NF-*κ*B significantly attenuated the CD137-CD137L induced increase of NF-*κ*B p65 and NFATc1. Inhibition of NF-*κ*B significantly attenuated the CD137-CD137L induced NFATc1 upregulation. Moreover, suppressing TRAF6/NF-*κ*B/NFATc1 significantly attenuated the agonist-CD137mAb induced increase of IL-2 and IL-6 in VSMCs. Thus, our data suggest that CD137-CD137L regulates NFATC1 expression through TRAF6/NF-*κ*B pathway in mouse VSMCs.

CD137 is a costimulator of T cells, and CD137 signaling has been reported to perform an important role in autoimmune disease, such as systemic lupus erythematosus, tumor, and atherosclerosis [4, 16]. Olofsson and his colleagues showed that CD137 was found to be expressed in human atherosclerosis and promoted plaque inflammation process development in hypercholesterolemic mice [3]. Our present study showed that CD137-CD137L interaction can regulate the expression of NFATc1 in ApoE^−/−^ mice spleen [11]. It is still unclear how CD137 signaling could regulate the expression of NFATc1. Recently, Sabbagh and his colleagues demonstrated that the tumor necrosis factor receptor-associated factor-1 [17, 18] may be the downstream signaling molecule of CD137 and TRAF1 phosphorylation on serine 139 modulated NF-*κ*B activity downstream of CD137 in T cells. Also McPherson et al. [19] discussed the role of TRAF1 in the alternative and classical NF-*κ*B pathway in T cells. A recent study showed that TRAF2 may also be involved in the activation of NF-*κ*B under CD137 signaling [20]. According to Hauer and colleagues [20], TRAFs are cytoplasmic adaptor proteins binding to various receptors of the TNFR family, such as CD40, CD137, and OX40. CD40 signaling may activate the downstream TRAF6 and mediate NF-*κ*B activation. They also found that TRAF6 knockout did not completely block NF-*κ*B signaling of the TNFRSFs. Furthermore, several studies showed that TRAF6/NF-*κ*B/NFATc1 signaling was an important pathway in osteoclast differentiation and bone resorption [12]. In this paper, we found that CD137 signaling affects the expression of NFATc1 in mice VSMCs through TRAF6/NF-*κ*B p65 pathway.

It is known that CD137 can be induced by cytokine mix (TNF-*α*, INF-*γ*, and IL-1*β*) in nonimmune cells, such as endothelial cells, VSMCs. Jeon et al. [16] also used this cytokine mix to induce the expression of CD137 in MOVAS cell line. In our studies, we found that TNF-*α* can stimulate the primary VSMCs to express the CD137 molecule, and we found the VSMCs may be influenced by three cytokines to study the downstream signaling. Most TNFRSF members (CD40, OX40, and CD137) require downstream adaptor proteins to induce intracellular signaling; TNFR-associated factors may be the most important adapter proteins. There are six TRAF members in cells. We all know that TRAF1, TRAF2, TRAF5, and TRAF3 may be involved in this pathway, but TRAF6, a key member of TRAF family, also participates in the activation of signaling in cells. The adapter protein TRAF6 is critical for mediating signal transduction. TRAF6 RING finger domain functions as an ubiquitin E3 ligase capable of generating nondegradative K63-linked ubiquitin chains. K63-linked autoubiquitination of TRAF6 is essential to form and activate a complex function involving the kinase TAK1 and its adapters, TAB1 and TAB2 [21]. The activity of TRAF6 has been demonstrated to require its autooligomerization and Lys-63-dependent polyubiquitination. Oligomerization is linked to its activity as an E3-ligase, leading to the activation of IKK [14, 22]. This finding indicated that autoubiquitination of TRAF6 is required for its activity toward TAK1 and IKK. IKK complex consists of three subunits, the catalytic subunits IKK*α* (IKK1) and IKK*β* (IKK2), and the regulatory subunit NEMO (IKK*γ*) [21]. Cell surface receptors initiate signaling cascades that converge on the activation of the inhibitor of *κ*B kinase (IKK) complex. IKK phosphorylation of inhibitor of *κ*B (I*κ*B) molecules promotes their degradation and releases NF-*κ*B (dimers of p65 and p50), which then translocates to the nucleus to promote transcription of target genes. NF-*κ*B, including p50, p52, p65, c-Rel, and RelB, is a nuclear transcription factor. NF-*κ*B signaling is involved in cell proliferation, inflammation, apoptosis, and growth [23]. In our study, agonist-CD137mAb was used to active CD137 signaling in VSMCs. The expression of TRAF6 and NF-*κ*B (p-p65) was increased simultaneously. Interestingly, we found that TRAF6 was still increased after p-p65 began to degrade. We believe that there are several possible explanations for this observation. Firstly, CD137 signaling activates the TRAF6/p-p65; p-p65 entered the nucleus and triggers the biological effect. Once p-p65 has transmitted the stimulatory signal from CD137 to its downstream target genes, its presence in the nucleus is no longer necessary and therefore subject to degradation. Secondarily, the biological effect triggered by p-p65 may promote the TRAF6 expression as a feedback mechanism. Thirdly, during the experiment, the VSMCs are under continuous inflammatory stimulation, which may further induce the expression of TRAF6.

In our study, we found that p-p65 and NFATc1 were inhibited when TRAF6 siRNA was added to the cells to knock down the expression of TRAF6. Although the reduced expression of NFATc1 in siTRAF6 group was significant compared with stimulated group, there are additional factors involved. CD137 signaling may be one of the pathways to activate the NFATc1, while other pathways also affect the expression. Also TRAF6 downregulation by siRNA is not very marked; this may affect the results. Transfection efficiency should be improved in the future experiment. When we blocked p-p65 using PDTC, we discovered that the expression of NFATc1 was inhibited. Our previous studies also proved this finding, and we found one of downstream target genes of CD137 was nuclear factor NFATc1, which can regulate the release of inflammation and amplify the inflammatory response in atherosclerosis. According to our results, the levels of IL-2 and IL-6 decreased when we suppressed NFATc1.

J. H. Kim and N. Kim [12] found that there were some key signal pathways in the progress of osteoclast differentiation. One of these pathways was TRAF6/NF-*κ*B/NFATc1 signal pathway; the pathway had an important role in signal transduction. In our studies, TRAF6 and NF-*κ*B p65 were involved in CD137 signaling. CD137 signaling may lead to the activation of TRAF6 by its ubiquitination and deubiquitination. The activated TRAF6 may lead to the activation of IKK, which may degrade I*κ*B from NF-*κ*B complex (including p65 and p50). Then, NF-*κ*B p65 was phosphorylated and entered into the nucleus from the cytoplasm [15], resulting in the transcription and translation of the target gene.

NFATc1 is the most expressed isoform in smooth muscle cells. Research showed that NFATc1 is an important gene in vascular calcification and inflammation. Recent studies demonstrated that NFATc1 plays an important role during the development of atherosclerosis [24]. NFATc1 signaling pathway is involved in the proliferation, inflammation, and calcification of VSMCs. NFATc1 was activated through dephosphorylation which allows it to enter the cell nucleus, thus producing many biological effects. But the exact mechanisms as to how the NF-*κ*B signaling pathway activates NFATC1 remained unclear and require our further study.

In summary, CD137 induced expression of NFATc1 in mice VSMCs was mediated by the TRAF6/NF-*κ*B pathway. Although we found that the proteins of TRAF6 and p-p65 were involved in CD137 signaling and may control the downstream molecule NFATc1, several issues need to be addressed in the future. We need to learn how CD137 signaling leads to the activation of TRAF6. Because of the low efficiency of siTRAF6, we should find the new way to knock out the TRAF6. We need to learn more about the signaling between NF-*κ*B p65 and NFATc1. At last, there may be other pathways involved in this process; therefore, the detailed mechanisms of atherosclerosis will continue to be exploited in the future. The influence of CD137 signaling on the expression of NFATc1 through TRAF6 and NF-*κ*B p65 may have extensive implications for the diagnosis and therapeutic intervention for a variety of proliferative cardiovascular diseases.
